# Polymorphisms in the Dopaminergic Receptor D3 Gene Correlate with Disease Progression Rate in Relapsing–Remitting Multiple Sclerosis Patients

**DOI:** 10.3390/genes15060736

**Published:** 2024-06-03

**Authors:** Marco Ferrari, Domizia Vecchio, Sandra D’Alfonso, Alessandra Gemma, Franca Marino, Cristoforo Comi, Marco Cosentino

**Affiliations:** 1 Center of Research in Medical Pharmacology, University of Insubria, 21100 Varese, Italy; agemma@uninsubria.it (A.G.); franca.marino@uninsubria.it (F.M.); marco.cosentino@uninsubria.it (M.C.); 2 Neurology Unit, Department of Translational Medicine, Maggiore Della Carità Hospital, University of Piemonte Orientale, 28100 Novara, Italy; domizia.vecchio@gmail.com (D.V.); cristoforo.comi@med.uniupo.it (C.C.); 3 Department of Health Sciences, Interdisciplinary Research Center of Autoimmune Diseases (IRCAD), University of Piemonte Orientale, 28100 Novara, Italy; sandra.dalfonso@med.uniupo.it; 4 Genetic Laboratory, Department of Translational Medicine, University of Piemonte Orientale (UPO), 28100 Novara, Italy

**Keywords:** polymorphisms, multiple sclerosis, dopamine receptors D3, MSSS

## Abstract

Background: Multiple sclerosis (MS) is a common chronic autoimmune disease of the central nervous system. In MS, disability progresses unpredictably. Dopamine (DA) is a modulator of immune functions, and compelling evidence supports its involvement in both pathogenesis and treatment of MS. Although single nucleotide polymorphisms (SNPs) in dopaminergic receptor (DR) genes have been extensively studied, their role in MS progression remains unexplored. Therefore, the aim of this explorative study is to investigate the potential association between functional SNPs in DR genes and MS progression. Methods: Caucasian patients with relapsing–remitting (RR) MS were enrolled, and disease progression assessed by the Multiple Sclerosis Severity Score (MSSS). Results: Out of the 59 RRMS patients enrolled, those with the G/G genotype for rs6280 and rs1800828 SNPs in DRD3 showed significantly higher MSSSs compared to those with ancestral and heterozygous genotypes. Conclusions: If confirmed in a larger prospective study, the reported findings could contribute to a better understanding of MS pathophysiological mechanisms, opening the way for the identification of marker(s) for assessing MS progression as well as novel therapeutic strategies. A personalized approach to MS management has the potential to improve the overall well-being of MS patients and alleviate the burden on their caregivers.

## 1. Introduction

Multiple sclerosis (MS) is the most common chronic autoimmune disease of the central nervous system. MS has an unknown etiology and develops through a highly heterogeneous and unpredictable course. MS is categorized into different forms; however, the most common of these is relapsing–remitting MS (RRMS), which affects 85% of MS patients [1].

The progression of MS is evaluated using various tools. The Expanded Disability Status Scale (EDSS) is a commonly used measure that assesses both neurological and physical impairments [2]. However, the EDSS does not account for disease duration. In contrast, the Multiple Sclerosis Severity Score (MSSS), a numerical score related to the EDSS, analyzes the rate at which disability evolves over time and provides a measure of MS progression. It allows for the stratification of patients based on disease duration and progression rate [3].

The neurotransmitter dopamine (DA) is an established modulator of immune functions [4,5,6], and compelling evidence supports its involvement in the pathogenesis of MS as well as in response to immunomodulatory drugs [4,5,6,7,8,9].

Dopamine acts on five dopaminergic receptors (DR): D1, D2, D3, D4, and D5. They are members of the G-protein-coupled receptor family and are divided into two families according to their pharmacological profile: “D1-like”, including D1 and D5, which activate adenylate cyclase, and “D2-like”, including types D2, D3, and D4, which inhibit adenylate cyclase [10]. Many different functional single nucleotide polymorphisms (SNPs) in dopaminergic receptor (DR) genes have been characterized in health and disease, specifically in many neurological diseases and psychiatric disorders, including schizophrenia, bipolar disorder, addiction, and Parkinson’s disease [11,12,13,14,15]. For a comprehensive review of the biological effects of SNPs in DR, see Magistrelli et al. [11]. Circumstantial evidence suggests that SNPs in DR genes affect the immune system, including circulating lymphocyte and CD4+ T lymphocyte counts [16], and a function of CD4+ T regulatory (Treg) cells [17]. Although these observations raise the possibility that SNPs in DRs may influence the clinical course of immune and inflammatory diseases like MS, their possible role in the development and progression of MS, however, has so far never been investigated.

For these reasons, in this explorative study, we assessed the possible association between selected SNPs in DR genes and MS progression.

## 2. Materials and Methods

### 2.1. Patients

This study included Caucasian patients with relapsing–remitting RRMS diagnosed according to McDonald’s criteria [18] who were originally enrolled from 2010 to 2013 in a cross-sectional multicenter study (PROGEMUS—PROgnostic GEnetic factors in MUltiple Sclerosis); they were regularly followed-up for at least 10 years after diagnosis.

Patients were recruited at the MS Centre, SCDU Neurology, “Azienda Ospedaliera Universitaria” Maggiore Della Carità, Novara, Italy. The study received approval from the Ethics Committee Ospedale Maggiore Della Carità, Novara (Study number 38/05, protocol # 156/CE (03.20.2009)). Informed consent was obtained from all participants.

MS progression was evaluated by MSSS, a probabilistic algorithm that incorporates the EDSS to calculate disease severity and duration. Patients were stratified according to MEDIAN MSSS (patients with MSSS ≤ 5 indicating mild disease, and patients with moderate/severe disease MSSS > 5) or according to the extremes of disease severity (EXTREME MSSS: patients with benign disease MSSS < 2.5, and patients with severe disease MSSS ≥ 7.5), as previously described [19].

The disease duration was defined as the time between the first symptom and the EDSS assessment, as described in Roxburgh [20].

### 2.2. Genotyping

We selected a panel of DR SNPs, giving priority to those with an expected frequency in Caucasian populations of at least 10%, with evidence of functional relevance, and/or that we showed in previous studies to be associated with clinical responses to dopaminergic agents [14,15]; for further details, see Table 1. Drawing from our previous experience [14,15], the choice of SNPs with high frequencies increases the probability of identifying potential differences in their frequency in relation to the relevant phenotypic aspects, even within a relatively small patient cohort. Furthermore, selecting SNPs with well-established biological effects enhances the likelihood of their contribution to determining patients’ phenotype. Genomic DNA was extracted from whole blood according to the standard protocols (QIAamp DNA Blood Mini kit, Qiagen, Mississauga, ON, Canada). The SNPs were identified with a real-time PCR system (Applied Biosystems, Foster City, CA, USA) by using TaqMan probe.

### 2.3. Statistics

Data are shown as the mean ± standard deviation (SD), unless otherwise stated. The statistical significance of the differences between groups was assessed by the Mann–Whitney U-test.

The χ^2^-test was used to assess the Hardy–Weinberg equilibrium in allele distributions. 

The influence of possible confounders (patient age and gender, age at onset, disease duration, BMI, ratio last ESDD/disease duration, and drug therapy) on the variable of interest was analyzed through univariate analysis based on a linear regression model. This analysis showed no associations between the variables listed above and the MSSS; therefore, they were not used for further multivariate analysis.

Differences in allele frequencies between groups were analyzed by the χ^2^-test for trends (or Fisher’s exact test, as appropriate). A one-way analysis of variance (ANOVA) test with a post-test for the linear trend was used to compare normally distributed variables, and the Kruskal–Wallis H test was used for non-normally distributed variables between the MSSS subgroups. The odds ratio (OR) with a 95% confidence interval (CI) was calculated using a recessive model (wild type/heterozygous vs. homozygous for SNP).

Statistical analyses were performed using GraphPad Prism version 5.00 for Windows (www.graphpad.com).

## 3. Results

### 3.1. Patients

A total of 59 patients with RRMS were enrolled in this study. For each patient, the age of disease onset, disease duration, last EDSS, and MSSS over a ten-year period were obtained from medical records. The detailed demographic and clinical features of the subjects included in the study are shown in Table 2.

### 3.2. Association between DR Genotypes and MSSS

All the alleles were in the Hardy–Weinberg equilibrium. 

**Figure 1:**
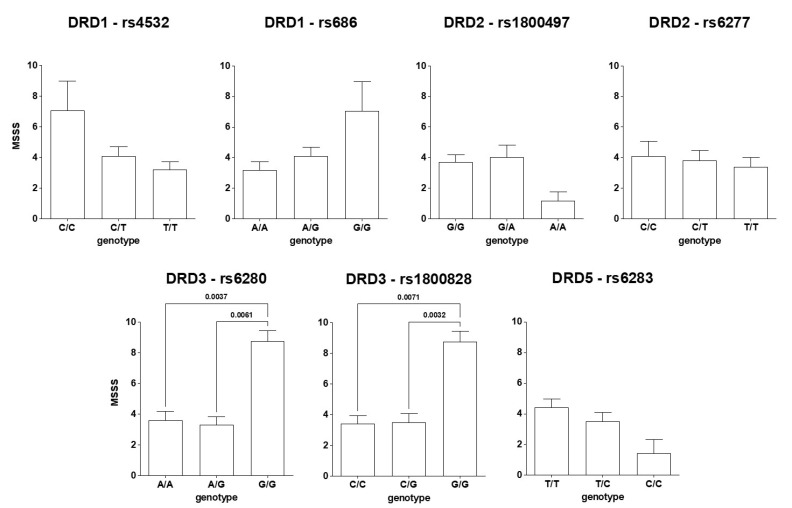
Relationship between SNPs in DR and MSSS.

Figure 1 shows that subjects with a G/G genotype for rs6280 and rs1800828 SNPs for DRD3 exhibited significantly higher MSSSs compared to those with ancestral and heterozygous genotypes. On the other hand, the SNPs in DRD1, DRD2, DRD4, and DRD5 showed no apparent association with MSSS, although a linear trend was found for DRD1 rs4532 and rs686 (r^2^ = 0.912; *p* = 0.1911).

When patients were stratified according to MEDIAN MSSS, we found a significant difference in frequencies in both rs6280 (*p* = 0.035) and rs1800828 SNPs in the different patient group. In particular, using Fisher’s exact test, we found a significantly higher SNP frequency in MS patients with an MSSS greater than five compared to those with an MSSS lower than five (*p* = 0.035). This difference became even more evident in patients stratified according to EXTREME MSSS (*p* = 0.009) (Table 3).

No difference was found between any SNPs included in the study for last EDSS or age at onset (Table S1).

## 4. Discussion

This explorative study provides evidence suggesting an association between the rs6280 and rs1800828 SNPs in DRD3 and MS progression.

Although some studies have provided important clues for the identification of the genetic role in both the causes of MS [21,22,23] and its progression [24], this is the first study providing evidence of the effects of SNPs in genes coding for DRs on the prediction of disease severity.

Growing evidence suggests that DRD2/3 agonists such as pramipexole improve MS symptoms [25], while the D2-like receptor antagonist L750667 exacerbates the disease condition [26]. Moreover, Cosentino and colleagues found a correlation between DRD3 and DRD5 mRNA expression levels, some immune cell subpopulations, and a risk of progression from clinically isolated syndrome (CIS) to MS within 12 months [27]. The same research group found that the treatment of RRMS patients with IFN-β for 12 months increased the mRNA levels of DRD5 (D1-like DR) in circulating PBMCs, while the DRD2 mRNA levels progressively decreased, and that higher mRNA levels for DRD5 may predict a subsequent response to IFN-β therapy in MS patients [28]. Finally, it has been found that, in drug-naive subjects affected by RRMS, Treg cells showed higher DRD5 mRNA expression levels, and treatment with IFN-β reduced this expression to values lower than in the Treg cells of healthy subjects [29]. The higher DRD5 expression suggests that these receptors may contribute to the suppression of regulatory functions of Treg cells, resulting in enhanced disease activity [29]. Together, these results seem to suggest that, in MS patients, a shift in balance from D2-like to D1-like DRs could be related to both response to therapy and disease progression [5]. This hypothesis is supported by the observations that DRD3-knockout mice exhibited an increased production of pro-inflammatory cytokines, neuro-inflammatory processes, and more rapid EAE progression compared to wild-type mice [9].

Considering the potential effects of an unbalance in the D1-like/D2-like DR ratio in the course of MS, it is not surprising that SNPs in DR could contribute to influencing the D1-like/D2-like DR ratio. In particular, our working hypothesis is that the presence of functional SNPs affecting the DRD3 function plays a role in the rapid disease progression observed in some MS patients. Of course, this hypothesis needs to be rigorously tested in future specific experiments, including the evaluation of DR mRNA expression levels and/or functional assays (such as cyclic AMP production) that measure the specific activity of these receptors.

We are aware that the reported results are limited by a low number of enrolled patients, the non-prospective design of the study, as well as a lack of information regarding the relationship between disease progression and immunomodulatory therapies. Regarding the study’s experimental design, it is crucial to acknowledge that evaluating MS progression over a minimum of 10 years in a prospective longitudinal study is inherently difficult to achieve in a prospective study setting. However, despite its limitations, if confirmed in a larger prospective study including healthy subjects as a control group and a greater number of SNPs in both DR and other genes, these findings could contribute to a better understanding of the pathophysiological mechanisms of MS and open the door for identifying novel therapeutic strategies. Moreover, our results could help in the identification of a new marker(s) for assessing MS progression. The early identification of individuals with a high risk of rapid MS progression, who face greater disability and require specialized care, could facilitate the early recognition of a patient’s medical and nursing needs. This, in turn, can enable the implementation of personalized treatments tailored to each individual’s disease severity. A personalized approach to MS management has the potential to improve the overall well-being of MS patients and alleviate the burden on their caregivers.

## Figures and Tables

**Table 1:** Selected SNPs. AFs, allelic frequencies (https://www.ncbi.nlm.nih.gov/snp/, accessed on 25 May 2024).

Gene	SNPs	Nucleotide Change	AFs %	Biological Effect
*DRD1*	rs4532	−48A>G	37	Reduced efficacy of pharmacological treatment in schizophrenia [11,14,15]
	rs686	62C>T	62	Increased gene expression
*DRD2*	rs1800497	2137G>A	39	Reduced receptor density in striated nucleus of healthy subjects [11,14,15]
	rs6277	957C>T	19	Reduced receptor expression in cortex and thalamus of healthy subjects [11,14,15]
*DRD3*	rs6280	25A>G	54	Reduced dopamine binding affinity [11,14,15]
	rs1800828	−712C>G	33	n/a
*DRD5*	rs6283	978T>C	38	n/a

**Table 2:** Demographic and clinical features of the study population.

Number of Subjects	59
Gender (male/female)	24/35
BMI	23.6 ± 10.5
Age (years, means ± DS)	54.9 ± 10.9
Age of disease onset (years, mean ± DS)	33.0 ± 10.5
Disease duration	15.5 ± 6.3
Average EDSS score (mean ± DS)	3.8 ± 2.6
Ratio last ESDD/disease duration	0.2 ± 0.1
Average MSSS (mean ± DS)	3.7 ± 3.0
Drug treatment	
no therapy	30
interferon-β	10
glatiramer acetate	7
azathioprine	2
mitoxantrone	1
siponimod	2
teriflunomide	2
alemtuzumab	1
cladribine	1
dimethyl fumarate	1
fingolimod	1
ocrelizumab	1

**Table 3:** Correlations between patient’s genotype and MS progression. * = χ^2^-test for trend; # = Fisher’s exact test, n., number of patients.

GENE	SNP	MEDIAN MSSS				EXTREME MSSS			
		MSSS < 5 n. (%)	MSSS > 5 n. (%)	*p* *	*p* #	MSSS < 2.5 n. (%)	MSSS > 7.5 n. (%)	*p* *	*p* #
*DRD1*	rs686								
	A/A	24 (62)	8 (40)	0.041	0.151	16 (59)	3 (40)	0.124	0.228
	A/G	15 (38)	10 (50)			11 (41)	4 (50)		
	G/G	0 (0)	2 (10)			0 (0)	1 (10)		
	rs4532								
	C/C	0 (0)	2 (10)	0.042	0.111	0 (0)	1 (10)	0.124	0.423
	C/T	15 (38)	10 (50)			11 (41)	4 (50)		
	T/T	24 (62)	8 (40)			16 (59)	3 (40)		
*DRD2*	rs1800497								
	G/G	29 (74)	13 (65)	0.827	0.544	19 (70)	6 (70)	0.263	1.000
	G/A	8 (21)	7 (35)			6 (23)	2 (30)		
	A/A	2 (5)	0 (0)			2 (7)	0 (0)		
	rs6277								
	C/C	8 (21)	4 (20)	0.920	1.000	5 (19)	2 (20)	0.389	0.412
	C/T	16 (41)	8 (40)			10 (37)	4 (60)		
	T/T	15 (38)	8 (40)			12 (44)	2 (20)		
*DRD3*	rs6280								
	A/A	17 (44)	9 (45)	0.396	0.035	12 (44)	3 (40)	0.050	0.009
	A/G	22 (56)	8 (40)			15 (56)	2 (20)		
	G/G	0 (0)	3 (15)			0 (0)	3 (40)		
	rs1800828								
	C/C	23 (59)	9 (45)	0.076	0.035	15 (56)	3 (40)	0.032	0.009
	C/G	16 (41)	8 (40)			12 (44)	2 (20)		
	G/G	0 (0)	3 (15)			0 (0)	3 (40)		
*DRD5*	rs6283								
	T/T	17 (44)	10 (50)	0.452	1.000	10 (37)	4 (50)	0.385	1.000
	T/C	20 (51)	10 (50)			15 (56)	4 (50)		
	C/C	2 (5)	0 (0)			2 (7)	0 (0)		

## Data Availability

The original contributions presented in the study are included in the article/Supplementary Materials; further inquiries can be directed to the corresponding author.

## Supplementary Materials

The following supporting information can be downloaded at: https://www.mdpi.com/article/10.3390/genes15060736/s1. Table S1. Relationship between SNPs in DR and age at onset, disease duration, EDSS.
