# Supplementary material for: Polymorphisms in the Dopaminergic Receptor D3 Gene Correlate with Disease Progression Rate in Relapsing–Remitting Multiple Sclerosis Patients
Source: Genes (Basel). 2024 Jun 3;15(6):736. doi: 10.3390/genes15060736 (PMC11203028; doi:10.3390/genes15060736)
Supplement: Supplementary file 1 [file genes-15-00736-s001.zip › genes-3006335-supplementary.pdf]

Table S1. Relationship between SNPs in DR and age at onset, disease duration, EDSS.

Gene	SNP	**Age at onset** (years)	**Disease duration** (years)	**EDSS*** (means ± SD)	**EDSS#** median (min-max)
DRD1	rs4532				
	A/A	36,0±8,5	19,0±7,0	3,6±2,6	2,5 (1,0-9,0)
	A/G	34,5±10,4	18,0±5,4	3,7±2,6	2,0 (1,0-8,5)
	G/G	31,6±10,9	17,0±1,3	6,8±1,8	6,7 (5,5-8,0)
	rs686				
	C/C	31,6±10,7	17,0±1,4	6,8±1,3	6,7 (5,5-8,0)
	C/T	34,5±10,2	18,0±5,3	3,7±2,5	2,0 (1,0-8,5)
	T/T	36,0±6,0	19,0±7,1	3,6±2,5	2,2 (1,0-9,0)
DRD2	rs1800497				
	G/G	33,8±10,9	17,7±4,6	3,7±2,6	2,2 (1,0-9,0)
	G/A	30,8±9,1	21,2±8,9	4,0±2,6	3,5 (1,0-8,0)
	A/A	35,0±8,0	14,5±2,5	2,0±0,5	2,0 (1,5-2,5)
	rs6277				
	C/C	31,2±8,5	19,9±6,9	4,0±2,5	3,0 (1,5-8,0)
	C/T	33,8±9,4	18,7±6,6	3,7±2,6	3,0 (1,0-8,5)
	T/T	33,2±12,4	17,5±5,2	3,7±2,6	2,0 (1,0-9,0)
DRD3	rs6280				
	A/A	32,3±10,8	19,1±6,2	3,8±2,5	2,5 (1,0-8,0)
	G/A	32,8±9,9	18,5±6,3	3,4±2,4	2,0 (1,0-8,5)
	G/G	45,5±2,5	13,3±0,9	6,8±2,4	6,8 (3,5-9,0)
	rs1800828				
	C/C	33,2±10,2	18,9±6,1	3,6±2,4	2,5 (1,0-8,0)
	C/G	31,8±10,5	18,5±6,5	3,6±2,5	2,0 (10-8,5)
	G/G	45,5±2,5	13,3±0,9	6,8±2,4	8,0 (3,5-9,0)
DRD5	rs6283				
	T/T	25,0±6,0	18,2±5,8	4,0±2,5	3,5 (1,0-9,0)
	T/C	34,4±10,4	19,2±6,5	3,6±2,7	2,0 (1,0-8,5)
	C/C	32,9±10,1	11,1±2,0	1,5±0,0	1,5 (1,5-1,5)
